# New roles for the de-ubiquitylating enzyme OTUD4 in an RNA–protein network and RNA granules

**DOI:** 10.1242/jcs.229252

**Published:** 2019-06-17

**Authors:** Richa Das, Lukas Schwintzer, Stanislav Vinopal, Eva Aguado Roca, Marc Sylvester, Ana-Maria Oprisoreanu, Susanne Schoch, Frank Bradke, Meike Broemer

**Affiliations:** 1 Ubiquitin Signaling Group, German Center for Neurodegenerative Diseases (DZNE), Sigmund-Freud-Str. 27, 53127 Bonn, Germany; 2 Axon Growth and Regeneration Group, German Center for Neurodegenerative Diseases (DZNE), Sigmund-Freud-Str. 27, 53127 Bonn, Germany; 3 Institute of Biochemistry and Molecular Biology, Core Facility Mass Spectrometry, University of Bonn, Nussallee 11, 53115 Bonn, Germany; 4 Institute of Neuropathology and Department of Epileptology, University of Bonn, Sigmund-Freud-Str. 25, 53127 Bonn, Germany

**Keywords:** De-ubiquitylating enzyme, Ubiquitin, Stress granule, RNA transport, Translation

## Abstract

Mechanisms that regulate the formation of membrane-less cellular organelles, such as neuronal RNA granules and stress granules, have gained increasing attention over the past years. These granules consist of RNA and a plethora of RNA-binding proteins. Mutations in RNA-binding proteins have been found in neurodegenerative diseases such as amyotrophic lateral sclerosis (ALS) and frontotemporal dementia (FTD). By performing pulldown experiments and subsequent mass spectrometry on mouse brain lysates, we discovered that the de-ubiquitylating enzyme OTU domain-containing protein 4 (OTUD4) unexpectedly is part of a complex network of multiple RNA-binding proteins, including core stress granule factors, such as FMRP (also known as FMR1), SMN1, G3BP1 and TIA1. We show that OTUD4 binds RNA, and that several of its interactions with RNA-binding proteins are RNA dependent. OTUD4 is part of neuronal RNA transport granules in rat hippocampal neurons under physiological conditions, whereas upon cellular stress, OTUD4 is recruited to cytoplasmic stress granules. Knockdown of OTUD4 in HeLa cells resulted in defects in stress granule formation and led to apoptotic cell death. Together, we characterize OTUD4 as a new RNA-binding protein with a suggested function in regulation of translation.

## Abstract (precis)

**Highlighted Article:** Novel functions for the de-ubiquitylating enzyme OTUD4 in RNA binding, stress granule formation and neuronal RNA transport granules are presented.

## INTRODUCTION

The flow of genetic information from gene to protein is grounded on production, processing, transport and translation of mRNA. More than 1000 RNA-binding proteins (RBPs) orchestrate these processes in mammalian cells (Castello et al., 2012; Gerstberger et al., 2014), providing ample opportunities for post-transcriptional gene regulation. Specialized RNA-binding domains usually mediate the RNA-binding ability of these proteins (Lunde et al., 2007). In addition, proteins with large stretches of low complexity or intrinsically disordered regions (IDRs) can also bind RNA (Castello et al., 2012; Kwon et al., 2013), extending the cellular repertoire of RBPs.

Intense research over the past years has characterized several types of RNA granules, which are membrane-less cellular compartments comprising multiple RNAs and associated proteins (Buchan and Parker, 2009). Complexes of protein(s) with RNA are described as ribonucleoproteins (RNPs). Proteins with IDRs or prion-like domains promote liquid–liquid phase separation processes, which are key to the formation of RNA granules (Hyman et al., 2014; Molliex et al., 2015; Weber and Brangwynne, 2012).

Neuronal RNA granules transport mRNAs from the cell body to axons and dendrites to enable local translation (Bramham and Wells, 2007; Glock et al., 2017; Kiebler and Bassell, 2006). mRNA transport and local translation is an essential feature of neurons, whose complex morphology requires the ability to react locally and rapidly to signaling cues. Neuronal RNA granules bind to microtubule motor proteins for directional transport (Czaplinski and Singer, 2006). For protein synthesis to occur locally, translation needs to be suppressed during transport until translation gets induced, for example, through activation of neuronal receptors (Fritzsche et al., 2013; Kiebler and Bassell, 2006).

Similar RNA granules, the so-called stress granules (SGs), form upon acute cellular stress. For example, oxidative stress, heat shock or proteasome inhibition induce SG formation in the cytoplasm of eukaryotic cells and this is linked to a block in translation initiation (Buchan and Parker, 2009). The main components of SGs are mRNAs, translation initiation factors, ribosomal subunits and a wide range of RBPs. If the stress persists only for a limited time, granule formation is reversible and can protect cells from stress-induced apoptosis (Arimoto et al., 2008; Kwon et al., 2007; Takahashi et al., 2013).

OTU domain-containing protein 4 (OTUD4) is a de-ubiquitylating enzyme (DUB) belonging to the ovarian tumor (OTU) family. OTUD4 is able to cleave ubiquitin chains of two different linkage types (Mevissen et al., 2013; Zhao et al., 2015, 2018). Only limited knowledge exists regarding its physiological function. OTUD4 regulates dorsoventral patterning in zebrafish (Tse et al., 2013), and contributes to the alkylation damage response (Zhao et al., 2015) and to IL1-β-dependent nuclear factor (NF)-κB signaling (Zhao et al., 2018). Interestingly, OTUD4 exerts its role in alkylation damage response with the help of two other DUBs, USP7 and USP9X, and relies on their catalytic activity. The intrinsic catalytic activity of OTUD4 seems dispensable in this context (Zhao et al., 2015).

Homozygous mutations in OTUD4 were found in a familial form of Gordon Holmes syndrome. Gordon Holmes syndrome is characterized by hypogonadotropic hypogonadism and ataxia. Interestingly, patients with mutations in OTUD4 and the ubiquitin ligase RNF216 also developed dementia in addition to the described symptoms (Margolin et al., 2013).

In light of these findings, we sought to address the role of OTUD4 in a neuronal context by identifying new interaction partners in mouse brain lysates by performing pulldown experiments. Strikingly, OTUD4 interacted with many RBPs and was able to interact with RNA itself. Under physiological conditions, OTUD4 was part of motile neuronal RNA granules. We also identified OTUD4 as a critical component of stress granules and showed that granule formation is impaired in the absence of OTUD4. In line with an RNA-dependent function of OTUD4, our results suggest that OTUD4 is involved in basal translation.

## RESULTS

### OTUD4 interacts with a network of RBPs

**Fig. 1.**
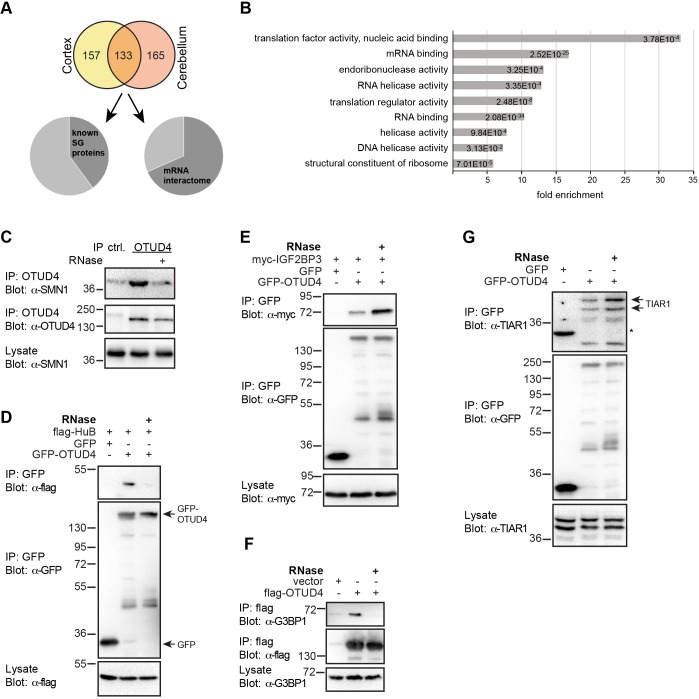
**OTUD4 interactome analysis places OTUD4 in a network of stress granule-associated proteins and RBPs.** (A) Identification of putative OTUD4-interacting proteins from mouse cerebellum and cortex lysate by mass spectrometry. Purified HA–OTUD4 or a HA-tagged control peptide were used as bait to pulldown interacting proteins from mouse brain lysates. Potential interactors were identified by mass spectrometry. An enrichment index for hits from at least two out of three experiments (per tissue) was calculated vs controls. Proteins with more than 30-fold enrichment over controls were considered as potential OTUD4 interactors. 290 proteins were identified in the cortex samples and 298 in the cerebellum samples with 133 proteins found in both tissues (Tables S1 and S2). 40% of these have been found in stress granules in previous studies (Jain et al., 2016; Markmiller et al., 2018) and 68% in mRNA interactomes (Beckmann et al., 2015). (B) Gene ontology analysis with the PANTHER GO slim tool (molecular function) revealed a strong enrichment of RNA-binding-related terms for the OTUD4-interacting proteins. Shown is the fold enrichment of terms compared to the mouse reference genome; depicted are all terms with an enrichment factor >5. The false discovery rate (FDR) for each of the terms is indicated in the respective bar. The analysis was performed for 298 potential interactors from cerebellum. (C–G) Co-IP experiments to check interactions of putative binding partners with OTUD4. (C) Endogenous OTUD4 was precipitated from HeLa cell lysates. Lysates were incubated at 37°C for 15 min in the absence or presence of 50 µg/ml RNase A. Co-precipitation of endogenous SMN1 was detected by western blotting. (D–G) HEK293T cells were transfected as indicated and lysates treated as above. Tagged OTUD4 was precipitated. Co-purification of exogenously expressed HuB (D) and IGF2BP3 (E) was confirmed, as well as of cellular G3BP1 (F) and TIAR (G). Treatment with RNase A (third lane of each panel) reduced or abolished the interaction with SMN1, HuB and G3BP1, demonstrating that these interactions are RNA dependent. In contrast, TIAR and IGF2BP3 seem to bind in an RNA-independent manner (* denotes cross-reaction of anti-TIAR-antibody with GFP). A representative of at least three independent experiments is shown for each IP.

To gain new insight into the biological function of OTUD4 with a focus on the nervous system, we sought to identify OTUD4-interacting proteins in mouse brain lysates. We expressed hemagglutinin (HA)-tagged OTUD4 or control vector in HEK293T cells and performed anti-HA-antibody affinity purification from HEK cell lysate. Beads with either bound HA–OTUD4 protein or HA–peptide as control were incubated with cerebellum or cortex lysate from mouse brain. We identified interacting proteins by mass spectrometric analysis and subjected them to stringent filtering to reveal OTUD4 interaction partners. We considered 298 proteins as highly enriched putative interactors in OTUD4 samples from cerebellum lysate and 290 from cortex lysate, of which 133 proteins were found in both tissues. (Fig. 1A; Tables S1–S3).

We performed a gene ontology (GO) analysis (cerebellum data set) with the PANTHER GO Slim tool (Mi et al., 2013; Thomas et al., 2003). Interestingly, the most highly enriched GO terms were all related to molecular functions linked to translation, nucleic acid binding and mRNA binding (Fig. 1B). In fact, we identified many known RBPs, for example, helicase MOV10 (MOV10), insulin-like growth factor 2 mRNA-binding protein 2 and 3 (IGF2BP2 and IGF2BP3) and fragile X mental retardation protein (FMRP, also known as FMR1) (Castello et al., 2012; Corbin et al., 1997; Feng et al., 1997; Gregersen et al., 2014; Meister et al., 2005). In line with this, 68% of the interactors shared between both tissues have been previously identified in mRNA interactome studies (Beckmann et al., 2015) (Fig. 1A). Furthermore, ∼40% of the putative interactors associated with stress granules in previous studies, including GTPase-activating protein-binding protein 1 (G3BP1) (Tourriere et al., 2003), T-cell-restricted intracellular antigen 1 (TIA1) and TIA1-related protein (TIAR, also known as TIAL1) (Kedersha et al., 1999), while others function in neuronal RNA granules, for example survival motor neuron protein 1 (SMN1) (Fallini et al., 2011; Zhang et al., 2006) and Staufen (Heraud-Farlow and Kiebler, 2014). Intriguingly, OTUD4 itself has been found in two mRNA interactome studies (Baltz et al., 2012; Beckmann et al., 2015) but the biological relevance or an RNA-related function of OTUD4 had not been investigated.

To validate some of the identified proteins as OTUD4 interaction partners, we performed co-immunoprecipitation (co-IP) experiments, focusing on known RBPs and stress granule proteins. We found that endogenous SMN1, FLAG-tagged HuB (also known as ELAVL2) and Myc–IGF2BP3 interacted with OTUD4 (Fig. 1C–E; Fig. S1). We also detected endogenous G3BP1 and TIAR protein, two core stress granule components, binding to OTUD4 in our co-IP experiments (Fig. 1F,G). Because these OTUD4-interacting proteins also bind RNA, we tested whether the observed co-IP depended on the presence of RNA. In fact, treatment of lysates with RNase prior to IP reduced or abolished the interaction of OTUD4 with SMN1, HuB and G3BP1, suggesting that OTUD4 binds to these proteins via RNA (Fig. 1C,D,F, right lane of each panel). In contrast, the interaction of IGF2BP3 and TIAR with OTUD4 was even enhanced after RNase treatment (Fig. 1E,G). This indicates that OTUD4 also undergoes direct protein–protein interactions with some binding partners and that these direct interactions were favored when RNA-dependent binding was abolished through RNase treatment.

### OTUD4 interacts with RNA

**Fig. 2.**
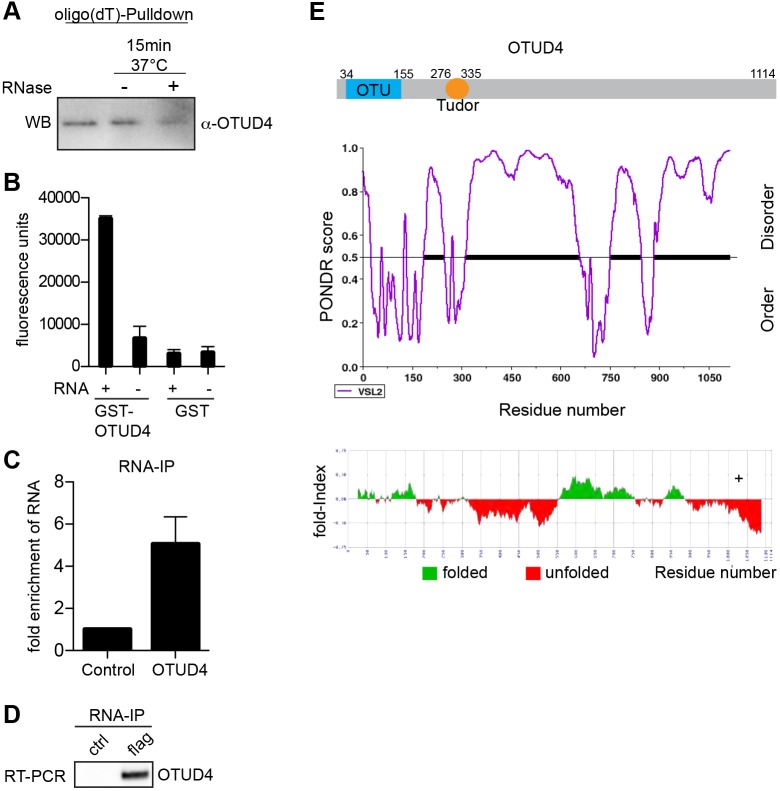
**OTUD4 interacts with RNA.** (A) Poly(A)-RNA was purified with oligo(dT)-beads from HEK293T cells and co-purified OTUD4 protein was visualized by anti-OTUD4 western blotting (WB). RNase treatment (50 µg/ml) reduced the amount of OTUD4 collected on oligo(dT)-beads, while control treatment at 37°C did not. A representative result of four independent experiments is shown. (B) Recombinant GST–OTUD4 binds RNA. GST–OTUD4 protein or GST alone were coupled to glutathione–Sepharose and incubated with biotinylated RNA or without RNA. Binding was detected by incubation with fluorescently labeled streptavidin. The mean±s.e.m. of three independent experiments is shown. (C) RNA-immunoprecipitation (RNA-IP) leads to enrichment of RNA with OTUD4. Anti-FLAG IP was performed from lysates of control (vector)- or FLAG–OTUD4-transfected cells. The amount of isolated RNA with OTUD4 is shown in relation to RNA purified in the control samples. Shown is the mean±s.e.m. from *n*=6 experiments. (D) Detection of OTUD4 mRNA bound to OTUD4. RNA-IP was performed from FLAG–OTUD4-transfected HEK293T cells under stringent conditions. RNA was extracted from IgG-control beads or anti-FLAG beads, and equal RNA volumes were used for RT-PCR reactions. To avoid overamplification of transfected OTUD4 RNA, primers for the 3′UTR of OTUD4-mRNA were chosen, which is not part of the overexpression construct. RT-PCR was performed from two independent RNA-IPs. (E) Domain structure of OTUD4. Top panel: OTUD4 contains an ovarian tumor (OTU) domain that provides activity to cleave ubiquitin chains as well as a putative Tudor-like domain. PONDR-VSL2 (middle panel) and foldIndex (bottom panel) algorithms predict that large portions of OTUD4 are disordered or unfolded.

**Fig. 3.**
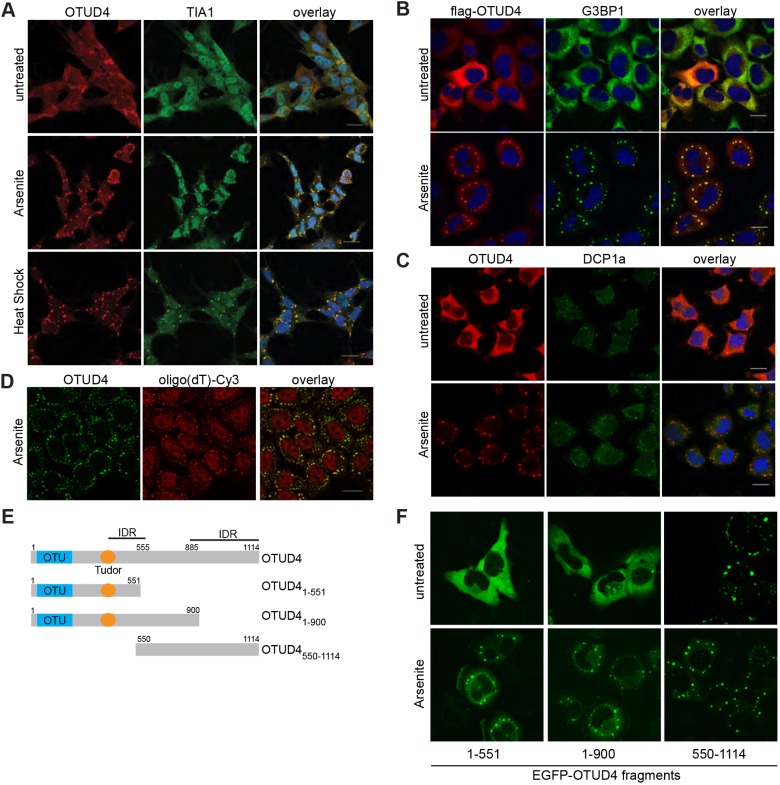
**OTUD4 is recruited to stress granules.** (A) Immunofluorescence of OTUD4 (shown in red) in SH-SY5Y cells that were either untreated, arsenite-treated (30 min, 0.5 mM) or heat-shocked (42°C, 1 h). Cells were co-stained for the stress granule marker protein TIA1 (green) and DAPI as a nuclear marker (blue). OTUD4 is redistributed to granular structures upon arsenite and heat-shock treatment. Granules also contain TIA1 and are considered as stress granules. Scale bar: 20 µm. The experiment was repeated two times. (B) Exogenously expressed FLAG–OTUD4 is recruited to stress granules in HeLa cells. Transfected cells were arsenite-treated (0.5 mM) for 40 min or left untreated and co-stained with anti-FLAG (red) and anti-G3BP1 antibodies (green). Nuclei are shown in blue (DAPI). Scale bar: 20 µm. The experiment was done at least three times. (C) OTUD4 granules do not colocalize with P-bodies. Shown is immunofluorescence of HeLa cells (untreated or 0.5 mM arsenite for 30 min) stained with anti-OTUD4 (red) and anti-DCP1a (green) antibodies. Scale bar: 20 µm. The experiment was performed two times. (D) OTUD4 granules contain mRNA. HeLa cells were treated with arsenite (0.5 mM) for 1 h. FISH was carried out with Cy3-labeled oligo(dT) (red), and cells were co-stained with anti-OTUD4 (shown in green). Scale bar: 20 µm. A representative image from four independent experiments is shown. (E) Scheme illustrating OTUD4 fragments used in F, numbers indicate amino acid borders of expression constructs. IDR, intrinsically disordered region. (F) HeLa cells were transfected with EGFP-tagged OTUD4 expression constructs as shown in E) and treated with arsenite (0.5 mM for 30 min) or left untreated to monitor intrinsic ability to form granules.

The following findings gave a strong hint towards an RNA-related function of OTUD4: (1) our OTUD4 interactome data showed a strong enrichment in RBPs (Fig. 1), (2) several interactions with RBPs were sensitive to RNase treatment (Fig. 1C,D,F) and (3) OTUD4 has been found in several mRNA interactome studies (Baltz et al., 2012; Beckmann et al., 2015). Therefore, we tested whether OTUD4 binds RNA and performed an oligo(dT)-pulldown experiment. Poly(A)-RNA was extracted from HEK293T cell lysates by using oligo(dT)-coated magnetic beads. Indeed, we confirmed co-purification of OTUD4 with poly(A)-RNA by western blot analysis (Fig. 2A). Treatment of the oligo(dT)-beads with RNase reduced the OTUD4 signal. This result suggests that OTUD4 binds to poly(A)-RNA extracted from cells, in line with proteomic data from mRNA interactome studies.

To consolidate this finding, we performed an *in vitro* assay using recombinant GST-tagged OTUD4 and *in vitro*-transcribed biotinylated RNA. Because specific RNA targets or sequence motifs have not been identified, we produced RNA from a GFP-encoding template. GST–OTUD4 or GST protein alone, as a control, were coupled to glutathione–Sepharose beads and incubated with biotinylated RNA or without RNA. Binding was detected with fluorescently labeled streptavidin. The fluorescence intensity in the presence of biotinylated RNA was more than 10-fold higher for GST–OTUD4 compared to the GST control, indicating that OTUD4 was able to bind RNA under these conditions (Fig. 2B).

Next, we performed IPs from FLAG–OTUD4-overexpressing HEK293T cells to test for co-purification of RNA from cells. We applied UV crosslinking to stabilize RNA–protein interactions, immunoprecipitated FLAG–OTUD4 and subsequently isolated the co-precipitated RNA from OTUD4 or control beads. These RNA-IPs showed a clear enrichment of RNA with FLAG–OTUD4 compared to that seen in control IPs (Fig. 2C).

As many RBPs bind to their own mRNA, we performed RT-PCR with the isolated RNA from RNA-IP experiments and gene-specific primers for the 3′UTR of OTUD4 mRNA. Clearly, OTUD4 interacted with its own mRNA (Fig. 2D). During the past years, it has become evident that protein–RNA interactions can not only be mediated by classical RNA-binding domains but can also be determined through other domains or through non-classical protein features such as IDRs, especially when they are enriched in the glycine, arginine and lysine residues (Castello et al., 2012; Järvelin et al., 2016; Kwon et al., 2013). OTUD4 carries an OTU domain, which classifies it as de-ubiquitylating enzyme, and a putative Tudor domain, but does not possess any classical RNA-binding domains (Fig. 2E, top panel). By using two different prediction tools, foldIndex (Prilusky et al., 2005) and ‘Predictor of Natural Disordered Regions’ (PONDR^®^; www.pondr.com), we noted that OTUD4 contains several stretches of unfolded or IDRs, which might mediate OTUD4–RNA interactions (Fig. 2E) (Castello et al., 2012).

### OTUD4 is recruited to stress granules

In eukaryotic cells, stress granules form upon short-term exposure to, for example, oxidative stress, heat shock or osmotic stress and contain mRNAs, stalled translation complexes and various RBPs (Buchan and Parker, 2009). Many of the newly identified OTUD4 interactors have been found in stress granules in previous studies (Jain et al., 2016; Markmiller et al., 2018; Youn et al., 2018). Interestingly, we detected OTUD4 in a network of these proteins even in the absence of stress (Fig. 1), indicating that at least some of the proteins that colocalize in stress-induced granules already associate in unstressed cells. This has also been observed in two recent studies (Markmiller et al., 2018; Youn et al., 2018). To test whether OTUD4 associates with stress granules, we treated SH-SY5Y neuroblastoma cells with sodium arsenite, which induces oxidative stress, or applied heat shock (42°C). While OTUD4 protein was evenly distributed in the cytoplasm in unstressed cells, we observed substantial recruitment of OTUD4 protein into cytoplasmic granular structures, which also contained the stress granule marker protein TIA1 (Fig. 3A; see Fig. S1D for OTUD4 antibody validation). Exogenously expressed FLAG-tagged OTUD4 was also recruited to stress granules following arsenite treatment, here detected by co-staining for the core stress granule protein G3BP1 (Fig. 3B).

Some of the constituents of stress granules are also present in another type of RNP granule, so-called P-bodies, which are responsible for mRNA decay (Buchan and Parker, 2009). We tested whether OTUD4-containing granules also show characteristics of P-bodies and co-stained with an antibody against the mRNA-decapping enzyme 1 (DCP1a), a protein generally present in P-bodies (Sheth and Parker, 2003; van Dijk et al., 2002). Clearly, arsenite-induced OTUD4-containing granules did not overlap with DCP1a-containing P-bodies, suggesting that OTUD4 is specifically present in stress granules (Fig. 3C). To determine whether OTUD4 granules also included RNA, we performed *in situ* hybridization with a fluorescently (Cy3)-labeled oligo(dT) probe to detect poly(A)-tails of mRNAs. Under non-treated conditions, the Cy3 signal in the cytoplasm of HeLa cells was diffuse, whereas upon arsenite treatment it showed a granular pattern (Fig. S1F). A clear overlap of the Cy3 signal with anti-OTUD4 antibody staining following arsenite treatment confirmed that these granules contained mRNA (Fig. 3D). We conclude that OTUD4 is recruited to stress granules. To get an idea which region of OTUD4 was required for granule recruitment or formation, we produced three truncated expression constructs of OTUD4 (Fig. 3E). OTUD4 contains two large stretches of IDRs (Fig. 2E), which might be of particular importance for RNA binding and phase separation processes. Interestingly, OTUD4^550-1114^ was the only tested construct that had a strong propensity to form granules (or aggregates) even in the absence of stress (Fig. 3F). In addition to its disordered character, the C-terminal part of OTUD4 contains stretches rich in the amino acid motifs RGG, RG, RS and GYSG, which have been previously found in other disordered RBPs (Castello et al., 2012). However, since all tested fragments were recruited into stress granules, we conclude that several regions contribute to stress granule recruitment and possibly also RNA binding.

### OTUD4 is part of neuronal RNA transport granules

**Fig. 4.**
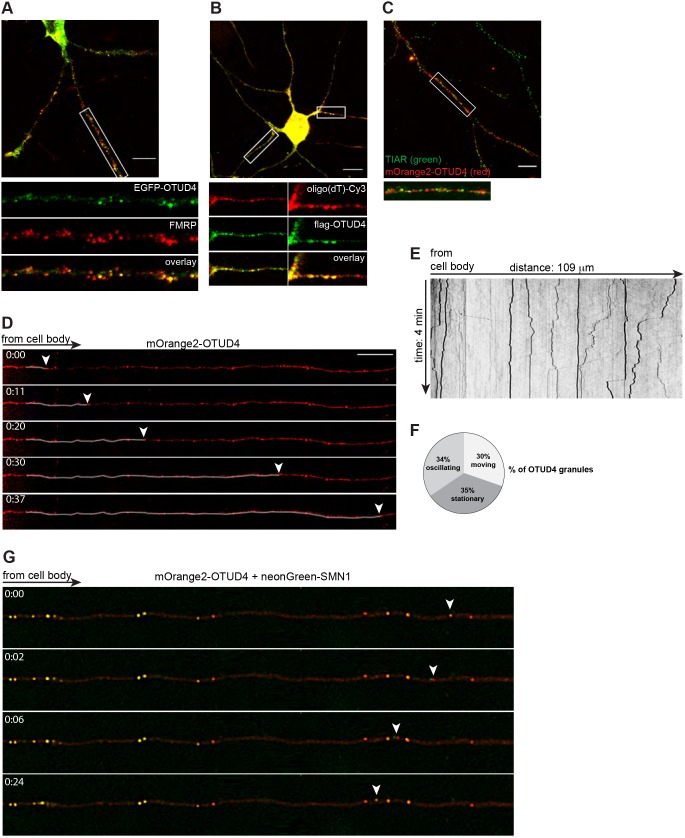
**OTUD4 is part of mobile neuronal RNA granules.** (A) Primary rat hippocampal neurons were transfected with EGFP–OTUD4 (green) and stained with anti-FMRP antibody (red) at days *in vitro* 4 (DIV4). Shown is an overlay of both channels, while the individual channels are shown for the enlargement of the boxed region (straightened). Magnification of the overlay image illustrates colocalization with FMRP. The EGFP control does not show neuronal granules (Fig. S3A). The experiment was performed five times. For quantification of colocalization, a total of 3204 OTUD4-containing granules were counted in ten neurons from two independent experiments. After filtering, 2433 OTUD4-positive granules were quantified as positive for a FMRP signal (76±11.8%, mean±s.d.). Scale bar: 10 µm. (B) OTUD4-positive granules contain RNA. Shown is a representative rat hippocampal neuron, transfected with FLAG–OTUD4. *In situ* hybridization with Cy3-oligo(dT) probe visualizes mRNA, and cells are co-stained with anti-FLAG antibody for FLAG-OTUD4 (DIV4). Magnifications of boxed regions (straightened) are shown below the overview picture. Note: to be able to visualize granules in the neurites the signal in the cell body had to be overexposed. The experiment was performed two times. A control Cy3-oligo(dT) hybridization of a neuron transfected with empty FLAG vector is shown in Fig. S3C. Scale bar: 20 µm. (C) mOrange2–OTUD4 does not colocalize with stress granule marker TIAR. Rat hippocampal neuron (DIV4), transfected with mOrange2–OTUD4 (red) and stained for TIAR (green). The image is an overlay of both channels. Scale bar: 5 µm. (D) Live-cell imaging of rat hippocampal neuron (DIV4), transfected with mOrange2–OTUD4. The ROI was straightened for illustration. Tracing shows anterograde movement of an OTUD4 granule (arrowhead), followed for 37 s. Scale bar: 20 µm. At least 30 neurons from three individual experiments were recorded. See also Movie 1. (E) Kymograph for the neurite presented in D. Vertical lines represent static granules, transversal lines demonstrate anterograde or retrograde movements of OTUD4 granules, recorded over a time course of 4 min. Line thickness correlates to granule size. (F) The mobility of 364 OTUD4-containing granules from a total of 13 neurons from three experiments was analyzed. Granule behavior was grouped in three classes, and results were visualized in a pie chart. Quantifications including standard deviation were: stationary, 35±22%; oscillating, 34±11%; mobile, 30±19%. (G) Hippocampal neuron (DIV4), transfected with mOrange2–OTUD4 and neonGreen–SMN1 for live-cell imaging. The ROI was straightened for illustration and granule movement was followed over 24 s. Most granules seem to contain both proteins. The white arrowhead denotes how a granule containing mOrange2–OTUD4 and neonGreen–SMN1 separates into two apparent units when moving retrogradely, with the neonGreen–SMN1 signal taking the lead. When the movement halts, mOrange2 and neonGreen signals overlap again (see Movie 2). At least 20 neurons from three independent experiments were recorded.

Neurons are highly specialized cells with unique morphology and function. To match these requirements, translation does not only occur in the cell body but also locally in axons, dendrites and synapses (for a review see Glock et al., 2017). In this way, the timely and regulated production of proteins at sites distant from the cell body is facilitated. Neuronal RNA granules are part of a transport machinery to carry mRNA from the cell body to distal neuronal processes (Bramham and Wells, 2007; Kiebler and Bassell, 2006) and share many features with stress granules. Some of the newly identified OTUD4-interacting proteins are involved in RNA transport in neurons, including Staufen, Pumilio 2, Purβ, FMRP (Table S1) and SMN1 (Fig. 1C) (Kanai et al., 2004; Zhang et al., 2003). Therefore, we examined whether OTUD4 protein – in addition to recruitment to stress granules upon acute cellular stress – was present in neuronal RNA granules under physiological conditions. Primary rat hippocampal neurons were transfected with EGFP–OTUD4 and imaged by confocal microscopy. EGFP–OTUD4 resided not only in the cell body but also showed prominent granular structures in proximal and distal parts of axons and in dendrites (Fig. 4A). In contrast, EGFP alone mainly localized in the cell body, with a very weak and diffuse pattern in the neurites (Fig. S3A). A protein which is well characterized for its role in neuronal granules and local protein synthesis is FMRP (Zalfa et al., 2006). Usually, neuronal RNA granules contain multiple RBPs in different combinations. We stained EGFP–OTUD4-expressing neurons with an anti-FMRP antibody to look for colocalization of EGFP–OTUD4 and FMRP (Fig. 4A). Quantification revealed that ∼76±11.8% (mean±s.d.) of OTUD4-containing granules also contained FMRP. Partial overlap between OTUD4 and FMRP was also observed with FLAG-tagged OTUD4 (Fig. S3B), while unfortunately no antibody was available to visualize endogenous OTUD4 in rodent neurons.

To elucidate whether the OTUD4 granules also contained RNA, we carried out *in situ* hybridization with a fluorescently (Cy3)-labeled oligo(dT) probe to detect poly(A)-tails of mRNAs (Fig. 4B), together with immunostaining for FLAG–OTUD4. We detected an overlap between OTUD4 and the Cy3 signal in granular structures in the neurites, indicating that OTUD4 granules contained mRNA. Of note, the Cy3 signal in the neurites was generally quite weak and, therefore, more OTUD4-positive granules were detected than Cy3-positive ones. This might be the reason why, in some regions, OTUD4 granules did not seem to be positive for mRNA, possibly due to a detection limit. Cy3-positive granules show a comparable pattern in control transfected neurons, ruling out that exogenous OTUD4 expression induces aberrant granule formation (Fig. S3C).

Furthermore, we stained mOrange2–OTUD4-transfected neurons for the stress granule marker protein TIAR, to test whether the observed granules were stress granules. Importantly, no overlap was observed between mOrange2–OTUD4 and TIAR in neurites in untreated neurons (Fig. 4C), indicating that the granular pattern of OTUD4 in neurons was not caused by an unwanted stress induction that could have originated from experimental conditions.

We showed that OTUD4 associates or colocalizes with proteins such as SMN1 (Fig. 1C) and FMRP (Fig. 4A), which are part of moving RNA-containing neuronal granules (Kanai et al., 2004; Zhang et al., 2003). This suggests that OTUD4-positive granules also might be mobile and actively transport mRNA along axons or dendrites. Live imaging of fluorescently tagged mOrange2–OTUD4 in rat hippocampal neurons showed numerous granules of heterogeneous size distributed along the axonal length. OTUD4 granules displayed three kinds of trafficking behaviors: namely they were (1) stationary, (2) had oscillatory movements, or (3) had directed movements in either anterograde or retrograde directions, or they had combinations of all three kinds (Fig. 4D,E; Movie 1). The majority of OTUD4 granules were relatively large in size and tended to be stationary. In contrast, smaller OTUD4 granules moved more continuously and faster. A kymograph plot (Fig. 4E) illustrates these granule behaviors: stationary granules are represented by a vertical line and granule movements cause deviations to the left (retrograde direction) or to the right (anterograde direction). Scoring the behavior of OTUD4-containing granules revealed the following distribution: 30% of granules were moving (either direction), 34% were oscillating and 35% were stationary over the recorded period of 4 min (Fig. 4F).

Because OTUD4 interacted with SMN1 in co-IPs and SMN1 has been well characterized in neuronal RNP granules (Fallini et al., 2011; Zhang et al., 2006), we co-expressed mOrange2–OTUD4 with neonGreen–SMN1 in hippocampal neurons for live-cell imaging. Most of the mOrange2–OTUD4 signal overlapped with that from neonGreen–SMN1 in granular structures in axons and dendrites, suggesting that these proteins are part of the same RNP granules. Tracking the movement of these granules by live imaging, we observed that most granules that contained both proteins were not moving, as has also been described for SMN1–HuD- and SMN1–Gemin2-containing granules (Fallini et al., 2011; Zhang et al., 2006). When starting to move, mOrange2–OTUD4 and neonGreen–SMN1 signals seemingly separated, which was likely caused by sequential imaging of the individual channels. Then they traveled closely together with the same speed and merged again when pausing (Fig. 4G; Movie 2).

Taken together, our results demonstrate that a fraction of OTUD4 is present in mobile neuronal RNPs, and that it might contribute to transport or regulation of mRNAs, maybe acting together with other RBPs such as FMRP or SMN1.

### Loss of OTUD4 impairs stress granule formation and leads to caspase activation

**Fig. 5.**
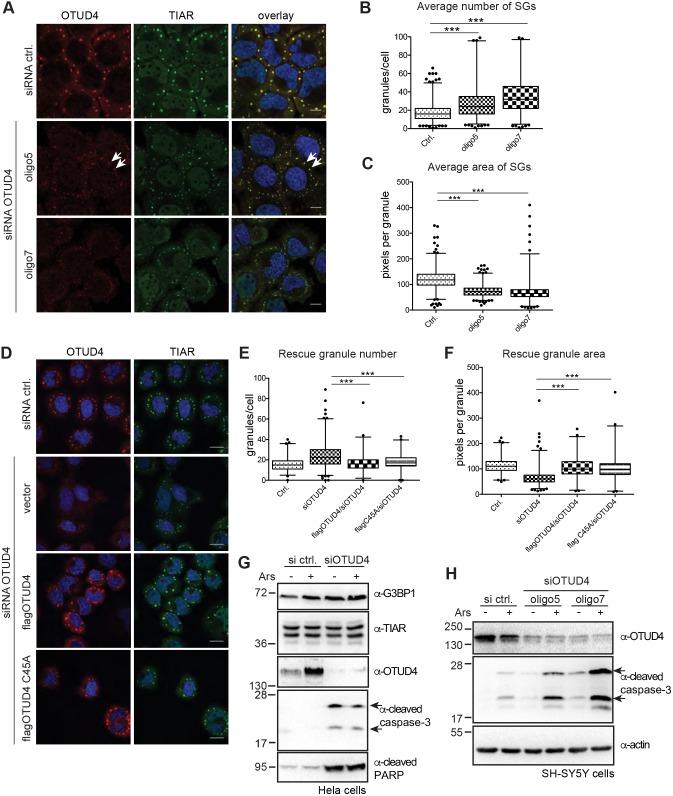
**OTUD4 is required for correct stress granule formation.** (A) Knockdown of OTUD4 decreases stress granule size and increases stress granule number. HeLa cells were transfected with two different siRNA oligonucleotides (oligo5 and oligo7) against OTUD4 or with control siRNA. OTUD4 and TIAR staining after 30 min arsenite treatment (0.5 mM) reveals differences in stress granule formation in the absence of OTUD4. White arrows indicate residual OTUD4 protein after knockdown, resulting in larger stress granules, resembling control siRNA-transfected cells. Scale bar: 10 µm. The experiment was repeated three times. (B) Quantification of the average number of stress granules per cell. Stress granules were scored in at least 200 cells per condition using CellProfiler software and the average number of granules per cell was depicted in a box plot. (C) Quantification of the average stress granule area. Granule area was determined with CellProfiler software as in B, and size distribution is presented as a box plot. (D) Re-introduction of siRNA-resistant OTUD4 rescues defects in stress granule formation. HeLa cells were transfected with FLAG–OTUD4 or its catalytic inactive mutant (C45A) 24 h after OTUD4 knockdown. Arsenite-treated cells (as in A) were stained with anti-OTUD4 and anti-TIAR antibodies. Scale bar: 20 µm. (E,F) Quantification of granule number and area in 55–100 FLAG–OTUD4-expressing cells per condition was performed as above. Wild-type and C45A-mutated OTUD4 reverse defects in granule formation. Cells with exogenous OTUD4 expression above endogenous levels were omitted for the analysis to exclude overexpression artifacts. In B,C,E,F, the box represents the 25–75th percentiles, and the median is indicated. The whiskers show the 1–99th percentiles and outliers are indicated. *P<0.05, **P<0.01, ***P<0.001 (B,E, quasi-Poissonian regression analysis; C,F, gamma regression analysis). (G) Analysis of changes in core stress granule proteins and apoptotic markers by western blotting. HeLa cells were transfected with OTUD4 siRNA or control siRNA with or without arsenite (30 min, 0.5 mM). Western blotting shows that G3BP1 and TIAR levels are unaffected by OTUD4 depletion. Depletion of OTUD4 leads to activation (cleavage) of caspase-3, as detected with anti-cleaved caspase-3 (Asp175) antibody and increased amounts of cleaved PARP, both indicating induction of apoptosis upon loss of OTUD4. The experiment was repeated three times. In D–G, oligo 7 was used for knockdown. (H) Knockdown of OTUD4 enhances the sensitivity of SH-SY5Y cells. SH-SY5Y cells were transfected with siRNA against OTUD4 or control siRNA as indicated. Cells were treated with arsenite (30 min, 0.5 mM) 24 h after siRNA transfection or left untreated. Cells were lysed 6 h later and levels of OTUD4, cleaved caspase-3 and actin were analyzed by western blotting. OTUD4-knockdown leads to increased caspase activation in response to arsenite treatment. The experiment was performed three times.

How does a cell react to the depletion of OTUD4? To address this question, we performed siRNA-mediated knockdown of OTUD4 in HeLa cells, which strongly reduced OTUD4 expression (Fig. 5A; Fig. S1D,E). After performing knockdown of OTUD4 with two different siRNA oligonucleotides, we induced stress granules with arsenite treatment. We observed that in the absence of OTUD4, stress granules, detected with anti-TIAR antibody, appeared to be much smaller in size but more numerous as compared to cells treated with control siRNA (Fig. 5A). Heat shock in OTUD4-depleted cells caused a similar phenotype (Fig. S2A). Quantitative assessment of average stress granule number per cell and average area per granule confirmed a significant difference between cells treated with control siRNA and cells lacking OTUD4, using two different siRNA oligonucleotides (Fig. 5B,C). Importantly, individual cells in which the knockdown of OTUD4 was incomplete, displayed stress granules of a size more similar to control cells (Fig. 5A, white arrows). The observed phenotype might be caused by a delay in stress granule development in OTUD4-knockdown cells. However, monitoring granule formation during longer arsenite treatment of up to 90 min revealed granules still remained small and fragmented, arguing against a changed time course of stress granule formation (Fig. S2B). The change in stress granule appearance upon OTUD4 depletion was also observed when cells were stained for G3BP1 (Fig. S2C), another core component of stress granules (Tourriere et al., 2003). This confirms that OTUD4 is not only required for proper formation of TIAR-containing granules but more generally affects stress granule formation.

To confirm that the observed phenotype is due to the knockdown of OTUD4, we re-introduced an OTUD4 expression construct that carried silent mutations in the siRNA-targeted sequence and can therefore be expressed in siRNA-treated cells. Only cells with expression levels of exogenous OTUD4 comparable to endogenous OTUD4 (in control cells) were considered for the analysis to minimize the occurrence of overexpression artifacts. Re-introduction of OTUD4 led to significant amelioration of the observed changes in granule size and number (Fig. 5D–F). Of note, overexpression of OTUD4 per se is not responsible for an increase in granule size (Fig. S3D).

Given that OTUD4 is a DUB, we hypothesized that OTUD4 potentially de-ubiquitylates one or several proteins within stress granules or during the process of granule formation. Several reports have indicated the presence of polyubiquitylated proteins in stress granules (Mateju et al., 2017; Turakhiya et al., 2018; Xie et al., 2018). By using an siRNA-resistant, catalytic inactive mutant (C45A; Zhao et al., 2015), we tested whether the catalytic activity of OTUD4 was required to restore normal stress granule formation in OTUD4-knockdown cells. Our results showed that catalytic activity of OTUD4 is dispensable for stress granule formation, as stress granules formed in a manner comparable to what was seen upon re-introduction of the wild-type (siRNA-resistant) OTUD4 (Fig. 5E,F). In addition, we generated an siRNA-resistant OTUD4 construct carrying the point mutation found in Gordon Holmes syndrome patients, G398V (Margolin et al., 2013), and tested this mutant with respect to stress granule formation. However, this point mutation in the IDR of OTUD4 did not affect the ability of OTUD4 to restore stress granule formation (data not shown).

How does OTUD4 contribute to stress granule formation? Does OTUD4 affect the levels of core stress granule proteins? We examined the abundance of G3BP1 and TIAR in lysates from control and OTUD4-siRNA-treated HeLa cells. G3BP1 protein levels mildly increased rather than decreased upon OTUD4 knockdown, while TIAR levels were unchanged (Fig. 5G), arguing against destabilization of these proteins when OTUD4 is depleted.

We noticed that siRNA-mediated knockdown of OTUD4 in HEK293T and HeLa cells led to increased cell death and analyzed lysates from control-siRNA- and OTUD4-siRNA-treated HeLa cells for markers of apoptosis. Indeed, we observed activation of caspase-3 and increased cleavage of PARP, a cellular caspase substrate, after 48 h of OTUD4 knockdown (Fig. 5G). This indicates that loss of OTUD4 triggers apoptotic cell death. We did not observe an increased additional sensitivity of OTUD4-depleted HeLa cells against stress induction (Fig. 5G, with and without arsenite). In contrast, neuroblastoma SH-SY5Y cells depleted of OTUD4 showed a much stronger activation of caspase-3 in response to arsenite treatment compared to control cells (Fig. 5H). This suggests that lack of OTUD4 and an altered stress granule response is detrimental for cell survival under stress conditions in certain cell types.

### Linking OTUD4 with the translation machinery

**Fig. 6.**
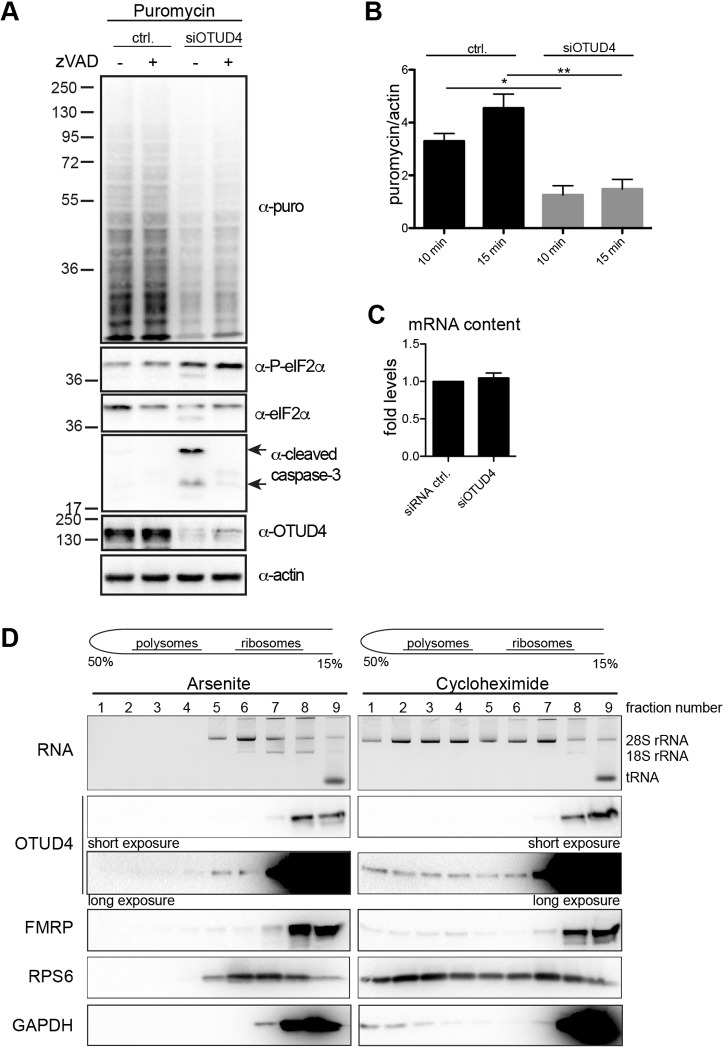
**OTUD4 associates with polysomes and loss of OTUD4 impairs protein translation.** (A) Loss of OTUD4 impairs protein translation. Control siRNA- or OTUD4 siRNA (oligo7, siOTUD4)-transfected HeLa cells were treated with caspase inhibitor z-VAD-fmk (24 h, 20 µM, starting 24 h after siRNA transfection) to control for caspase-related effects, or were left untreated. 10 µg/ml puromycin was added for 15 min to label newly synthesized proteins. Protein lysates were analyzed by immunoblotting for puromycylated proteins and levels of phosphorylated (P)-eIF2α, eIF2α, cleaved caspase-3, OTUD4 and actin. Anti-puromycin blot revealed lower levels of puromycin-labeled proteins in OTUD4 knockdown cells, indicating reduced translation in the absence of OTUD4. Phosphorylation of eIF2α was increased in OTUD4 knockdown cells. The observed effects were not blocked by caspase inhibition. A representative of at least three independent experiments is shown. (B) Puromycylation was performed for 10 or 15 min in control or siOTUD4 (oligo7)-transfected HeLa cells 48 h after transfection. Lane intensities were quantified using Fiji software and normalized to actin levels. The mean±s.e.m. of *n*=4 experiments is indicated. *P<0.05, **P<0.01 [paired *t*-test; *P*-values were 0.0219 (10 min puromycin) and 0.0036 (15 min puromycin)]. (C) The cellular mRNA content is not affected by OTUD4 knockdown. Poly(A)-RNA was extracted from HeLa cells transfected with control siRNA or OTUD4 siRNA. The mRNA content of siOTUD4-transfected cells is shown as fold level compared to that in control cells (set at 1). The mean±s.e.m. of *n*=3 experiments is shown. (D) OTUD4 is present in polysome-containing fractions. HeLa cells were either treated with arsenite (0.5 mM, 30 min), leading to polysome disassembly, or with cycloheximide (100 µg/ml, 15 min) to conserve mRNA–ribosome complexes. Lysates were subjected to sucrose gradient fractionation (15–50%) and individual fractions were divided for RNA preparation or for protein precipitation and subsequent immunoblot analysis. The approximate sedimentation behavior of polysomes and individual ribosomes, as judged by RNA profile and ribosomal protein S6 (RPS6) location, is indicated. Upon arsenite treatment, OTUD4 was found in fractions 5–9, while under cycloheximide conditions, it was also found in the putative polysome-containing fractions (2–4). For comparison, protein profiles of FMRP, known to associate with polysomes, RPS6 as ribosomal marker and GAPDH as a protein presumably unrelated to translation, are shown. A representative of three experiments is shown.

Our data indicate that OTUD4 is a cytoplasmic RBP. An important role of RBPs in the cytoplasm is the regulation of translation. A well-established assay to monitor translation is the ‘SUnSET’ assay (Schmidt et al., 2009), using the incorporation of puromycin, which mimics an aminoacyl-tRNA, into nascent polypeptides to compare the rate of protein synthesis under different conditions. Thus, the rate of puromycin incorporation directly reflects protein synthesis activity. Puromycylated proteins are subsequently detected by western blotting. To test whether OTUD4 was required for translation, we knocked down OTUD4 in HeLa cells and added puromycin for 15 min. OTUD4 knockdown (using oligo7) led to strongly reduced puromycin incorporation, as shown in an anti-puromycin antibody western blot (Fig. 6A). Quantification of puromycin signals and normalization to actin levels revealed a significant reduction of puromycin incorporation in OTUD4-knockdown cells (Fig. 6B). As we had observed increased caspase activation and apoptosis of cells upon OTUD4 knockdown (Fig. 5G,H), we used the pan caspase-inhibitor z-VAD-fmk to test whether the reduction in translation was a consequence of caspase activation (Fig. 6A). However, caspase inhibition did not restore protein translation, indicating that OTUD4 might play a direct role in the regulation of translation. Important regulators of eukaryotic translation are the eukaryotic translation initiation factors (eIFs). Phosphorylation of eIF2α (eIF2S1) by stress-sensing kinases occurs in many cases together with or upstream of translational arrest, and blocks the formation of preinitiation complexes required for translation initiation (Holcik and Sonenberg, 2005; Hinnebusch, 1994). We performed western blot analysis of lysates from OTUD4-knockdown cells using antibodies against phosphorylated eIF2α. Knockdown of OTUD4 enhanced eIF2α phosphorylation (Fig. 6A), which goes in line with reduced translation in these cells. Importantly, phosphorylation of eIF2α upon loss of OTUD4 was also not a consequence of caspase activation in these cells. Treatment with the pan caspase inhibitor z-VAD-fmk did not reverse the effect (Fig. 6A). Interestingly, we observed a low level of caspase-dependent cleavage of eIF2α in OTUD4-knockdown cells. Caspase-dependent cleavage of eIF2α has been described previously (Marissen et al., 2000) but blocking this event by caspase inhibitor z-VAD-fmk did not alter the translational reduction in OTUD4 knockdown cells (Fig. 6A).

A reduced rate of translation could be caused by an overall diminished mRNA content upon knockdown of OTUD4. However, isolation of poly(A)-RNA and quantification revealed no difference between control and OTUD4-knockdown cells regarding their mRNA content (Fig. 6C).

To further investigate the connection between OTUD4 and translation regulation, we performed sucrose gradient fractionation. Actively translating mRNA, polysomes and associated proteins can be distinguished from individual ribosomes and unbound mRNAs due to their sedimentation profile. HeLa cells were either treated with arsenite to block translation and induce polysome disassembly, or with cycloheximide, which conserves mRNA–polysome complexes. Lysates were subjected to gradient fractionation by performing ultracentrifugation, and the collected fractions were divided for RNA extraction and immunoblot analysis. The RNA profile, illustrated through 28S and 18S (minor) rRNA, showed a clear difference between arsenite- and cycloheximide-treated samples (Fig. 6D), which may be explained by the absence of polysomes upon arsenite treatment. Western blotting of the protein fractions containing OTUD4 also showed similar differences between both conditions. Most of OTUD4 protein was found in the top fractions (8 and 9) of the gradient, where most cytoplasmic proteins migrate. But a small fraction of OTUD4 co-sedimented with polysomes, mainly present in fractions 2–4 in the cycloheximide-treated set of samples, according to the rRNA profile. For comparison, fractionation profiles are also shown for FMRP [previously shown to co-fractionate with polysomes (Napoli et al., 2008)], ribosomal protein S6 (RPS6) as marker for ribosomes and GAPDH as cytoplasmic protein not related to translation. A small percentage of GAPDH was present in fraction 1, unrelated to polysomes, while OTUD4, in contrast, was distributed into the polysome-containing fractions 2–4. Taken together, the fact that OTUD4 co-migrates with polysome-containing fractions and that this is abrogated upon arsenite treatment further supports our findings for a potential role in regulation of translation.

Together, our data strongly link OTUD4 with mRNA-binding RNP granules and the translation machinery.

## DISCUSSION

Previous work has described a role of the DUB enzyme OTUD4 in dorsoventral patterning in zebrafish (Tse et al., 2013), in the alkylation damage response (Zhao et al., 2015), in IL1-β-dependent NF-κB signaling (Zhao et al., 2018) and in antiviral signaling (Liuyu et al., 2018). Our study reveals that OTUD4 is an RBP, is present in neuronal RNA granules and plays a role in translation regulation. Importantly, OTUD4 was required for proper formation of cytoplasmic stress granules upon acute cellular stress. Loss of OTUD4 led to apoptotic cell death. We will now discuss how our work changes the perspective on OTUD4 with respect to stress granule formation, granule transport in neurons, (local) translation and regarding its potential role in neurodegenerative diseases.

### OTUD4 is required for stress granule formation

Lack of OTUD4 during acute oxidative or heat stress led to characteristic changes in stress granule appearance: granules were smaller in size but much more numerous than in control cells. Given the high number of proteins and multivalent interactions that are involved in stress granule formation, it is an intriguing finding that knockdown of a single protein can alter the overall appearance of these granules. Interestingly, cells depleted for G3BP1 or TDP-43 display similar phenotypes (Aulas et al., 2015; Aulas et al., 2012) and OTUD4 interacted with both proteins. Granule formation is a dynamic process during which a stable core, consisting of mRNAs and bound proteins, forms. In a second phase, a so-called shell of more loosely connected proteins grows (Jain et al., 2016; Wheeler et al., 2016). While stress granules still form in the absence of OTUD4, they remain small and fragmented, suggesting that fusion or maturation of granules or the association of further shell proteins might be impaired.

### Does OTUD4 de-ubiquitylate specific proteins in RNA granules?

An obvious question arising from this finding is how does OTUD4 influence granule formation and whether the catalytic activity of OTUD4 is required. DUBs play pleiotropic roles in the regulation of cellular processes (Clague et al., 2013; Reyes-Turcu et al., 2009). When DUBs remove ubiquitin chains from other proteins, they can for example alter protein stability, protein localization or the function of their substrates. Surprisingly, we found that a catalytic inactive OTUD4 mutant behaved like the wild-type protein and restored normal granule formation upon knockdown of OTUD4. However, we cannot rule out that OTUD4 nevertheless de-ubiquitylates one or several proteins within stress granules, with implications only for these individual proteins but not for the process of stress granule formation altogether. Several other DUBs are part of stress granules, including USP10 (Kedersha et al., 2016; Takahashi et al., 2013), its yeast homologue Ubp3 (Nostramo et al., 2016), USP5 and USP13 (Xie et al., 2018). So far, no stress granule-specific substrate proteins of these DUBs have been identified, and diverging results exist for requirement of catalytic activity for the assembly or disassembly of granules (Nostramo and Herman, 2016; Xie et al., 2018). Identification of ubiquitylated proteins within stress granules will help to assess whether OTUD4 or other DUBs possess specific substrates within stress granules or whether they exert ubiquitin-independent roles in this context. Of note, OTUD4 also seems to act independently of its catalytic activity in response to alkylation-induced DNA damage (Zhao et al., 2015).

We detected OTUD4 not only in cytoplasmic stress granules but also in neuronal RNA transport granules. Knowledge regarding specific ubiquitylation and de-ubiquitylation events in neuronal RNA granules is also rather scarce, and it remains to be elucidated whether OTUD4 exerts DUB activity in neuronal RNA granules.

### OTUD4 is part of neuronal RNA transport granules

OTUD4 granules showed movements in anterograde and retrograde directions along neurites. Motor proteins usually drive these kinds of movements along microtubules (Bramham and Wells, 2007; Kiebler and Bassell, 2006) and it will be interesting to test the requirement of these components for OTUD4 granule mobility. As OTUD4 is part of SMN1-containing granules, it is possible that SMN1 recruits OTUD4 to these transport granules, as has been demonstrated for other proteins (Fallini et al., 2011; Zhang et al., 2006). The fact that OTUD4 granules actively move along neurites underlines that these are functionally active entities and not mere aggregates. Future work will be needed to investigate the function of OTUD4 in these granules, for example regarding local protein synthesis and synaptic plasticity.

### Potential involvement of OTUD4 in translation regulation

Several of our results hint towards an involvement of OTUD4 in translation-related processes. Translation of mRNAs transported in dendrites and axons is highly regulated, and it is tempting to speculate about a translation-related role of OTUD4 in neuronal granules. This might depend on the DUB activity of OTUD4, which might act to, for example, fine-tune levels of proteins that directly regulate translation. Intriguingly, the dendritic levels of FMRP, an interactor of OTUD4 and suppressor of mRNA translation during transport, can be regulated by ubiquitylation and subsequent degradation upon stimulation of the metabotropic glutamate receptor (Hou et al., 2006; Nalavadi et al., 2012). Alternatively, the impact of OTUD4 on translation could be due to its direct interaction with mRNAs, or through regulation of translation initiation. In line with a potential role in translation initiation, we found several eIFs in our OTUD4 interactome study (eIF3, eIF4A, eIF4E, eIF4G; Tables S1–S3). In addition, previous proteomic studies detected these proteins as potential OTUD4 interactors (Sowa et al., 2009; Zhao et al., 2018).

### RBPs and neurodegenerative diseases

RBPs play important roles in neurodegenerative diseases. Mutations in TDP-43 or FUS lead to ALS/FTD (Lagier-Tourenne et al., 2010; Vance et al., 2009), while mutations in Ataxin-2 can cause spinocerebellar ataxia type 2 or ALS (Elden et al., 2010; Gispert et al., 1993). These RBPs frequently contain intrinsically disordered regions and prion-like domains, promoting self-aggregation of these proteins. This might be the reason why they are also found in protein aggregates in sporadic forms of neurodegenerative diseases (Gitler and Shorter, 2011; Li et al., 2013). Furthermore, ALS-linked mutations in low complexity domains of TIA1 and hnRNPA1 lead to disturbed stress granule dynamics and impaired protein translation, causing chronic cellular stress (Kim et al., 2013; Mackenzie et al., 2017).

Impressive recent work has shown that reducing levels of TIA1 or ataxin-2 delays disease progression in models for Alzheimer's disease (AD) or ALS, respectively (Apicco et al., 2018; Becker et al., 2017), further supporting the importance of RBPs in neurodegenerative diseases. Of note, we found TDP-43, FUS, TIA1 and ataxin-2 in our interactome data for OTUD4.

Homozygous mutations in the *OTUD4* gene have been found together with mutations in a ubiquitin-E3-ligase in a familial form of Gordon Holmes syndrome, leading to hypogonadotropism, ataxia and dementia (Margolin et al., 2013). Interestingly, this point mutation lies within the IDR of OTUD4, and disease-relevant mutations in other proteins have often been found in IDRs, for example in FUS (Vacic et al., 2012; Vance et al., 2009). A thorough examination of potential functional consequences of this mutation is warranted in the future.

Taken together, our work identifies exciting new functions of OTUD4 and opens up new lines of research regarding its RNA-related roles and the function of a DUB enzyme in translation regulation and RNA granules.

## MATERIALS AND METHODS

### Chemicals and antibodies

All reagents/chemicals were obtained from Carl Roth or Sigma-Aldrich, unless stated otherwise. Cell culture reagents were from Gibco/Thermo Fisher Scientific. Complete protease inhibitor was from Roche. RNaseOUT ribonuclease inhibitor was from Invitrogen. The following primary antibodies were used: anti-β-actin (1:5000, Abcam, ab8224), anti-cleaved-caspase-3 (Asp175) (1:1000, Cell Signaling, #9661), anti-cleaved-PARP 4B5BD2 (1:1000, Abcam, ab110315), anti-Dcp1a (IF 1:100, Novus Biologicals, H00055802-M06), anti-eIF2a (1:1000, Cell Signaling, #9722), anti-P-eIF2a (1:1000, Cell Signaling, #9721), anti-FLAG M2 (WB, 1:5000; IF, 1:500, Sigma, F1804), anti-FMRP (WB, 1:1500; IF, 1:200, Abcam, ab17722 or 4G9, Thermo Fisher Scientific MA515499), anti-GFP (1:5000, Abcam, ab13970), anti-G3BP1 (WB, 1:1500; IF, 1:250, Abcam, ab181150), anti-GAPDH 14C10 (1:1000, Cell Signaling Technology, #2118), anti-HA 3F10 (1:2000, Roche, 11867423001), anti-HA (1:1000, Santa Cruz Biotechnology, SC-805), anti-c-myc 9E10 (1:1500, Life Technologies, 132500), anti-OTUD4 (WB, 1:1500; IF, 1:100 Sigma, HPA036623; and 1:3000, Bethyl Laboratories, A304-605A), anti-OTUD4 (1:2000, custom-made rabbit polyclonal, generated against human recombinant OTUD4 amino acids (aa) 1–615], anti-puromycin 12D10 (1:2500, Millipore, MABE343), anti-S6 Ribosomal Protein 5G10 (1:1000, Cell Signaling Technology, #2217), anti-SMN1 (WB, 1:2000; IF, 1:200; BD Biosciences, 610646), anti-TIA1 (1:500, Santa Cruz Biotechnology, sc1751) and anti-TIAR (WB, 1:1000; IF, 1:100; BD Biosciences, 610352).

### DNA constructs

Human OTUD4 cDNA coding for isoform 4 (1114 aa) was cloned in pEGFP-C3 (Clontech), pCMV-3Tag-6 (Agilent Technologies), pN3HA (kindly provided by Christoph Thiele, University of Bonn, Germany) or pGEX-6P-1 (GE Healthcare). The catalytically inactive mutant OTUD4 C45A was produced by site-directed mutagenesis (SDM). siRNA-resistant OTUD4 constructs were made by introducing four silent mismatches (bold) in the siRNA targeting region by using the following primer sequence (fwd) for SDM: 5′-GGGAACCAAATGT**C**TC**C**CC**A**TCACA**G**GTAACAGAAAATAATTTTC-3′.

GFP-SMN1 was from Addgene, deposited by Greg Matera (Addgene #37057, Shpargel and Matera, 2005), pFRT-TODestFLAGHA_HuB (Addgene #65755) and pDESTmycIGF2BP3 (Addgene #19879; Landthaler et al., 2008), deposited by Thomas Tuschl.

SMN1 was subcloned into pmNeonGreen-C1 (Gentaur Europe BVBA). The resulting mNeonGreen–SMN1 cassette was then excised and subcloned into a modified p-βactin vector (Kapitein et al., 2010), kindly provided by Casper Hoogenraad (Utrecht University, The Netherlands). For live-cell imaging, OTUD4 was cloned into p-βactin-mOrange2-C1 (mOrange2 was amplified from mOrange2–EB3-7, Addgene #57953, a deposited by Michael Davidson).

### Cell culture, transfections and stress granule induction

All cell lines were originally acquired from ATCC and regularly mycoplasma-checked. HEK293T, HeLa and SH-SY5Y cells were cultured in Dulbecco's modified Eagle medium (DMEM) supplemented with 10% fetal bovine serum and 50 units/ml penicillin and 50 µg/ml streptomycin (medium and supplements from Gibco) at 37°C and 5% CO_2_. Transfections of HeLa cells with expression plasmids were performed using Effectene Transfection Reagent (QIAGEN) according to the manufacturer's instructions. HEK293T cells were transfected by using the calcium phosphate method.

siRNA transfections were performed with Lipofectamine RNAiMAX (Thermo Fisher Scientific) according to manufacturer's instructions for 48 h with Hs_OTUD4_7 FlexiTube (oligo7) siRNA unless stated otherwise, Hs_OTUD4_2 FlexiTube (oligo2), and Hs_OTUD4_5 FlexiTube (oligo5) siRNA. Controls were transfected with AllStars Negative Control siRNA (all siRNAs were from QIAGEN).

To induce stress granules, cells were incubated with 0.5 mM sodium arsenite for the indicated time at 37°C. For heat-shock experiments, cells were placed at 42°C for 1 h.

### Primary neuronal culture and transfections

Primary hippocampal neurons were isolated and cultured from embryonic wild-type (E17) rats as described previously (DeHoop et al., 1997). Briefly, hippocampi were dissected, trypsinized (0.05% Trypsin-EDTA, Invitrogen) at 37°C for 15 min and washed in Hank's Balanced Salt Solution. All animal experiments were performed according to approved guidelines. For transfection, 3×10^5^ dissociated neurons were electroporated with the Amaxa Nucleofector system (Rat Neuron Nucleofector Kit, Lonza, program O-003) using highly purified DNA (EndoFree Plasmid Maxi Kit, QIAGEN). Cells were plated onto poly-D-lysine-coated coverslips in 6-cm petri dishes in minimum essential medium (MEM) with 10% heat-inactivated horse serum. The cells were maintained in 5% CO_2_ at 36.5°C. After 4 h, the coverslips were flipped onto a 6-cm dish containing astrocytes in MEM and N2 supplement.

For live-cell imaging experiments, 1.5×10^5^ neurons were transfected and plated onto poly-D-lysine-coated chamber slides (Ibidi). After 3 h, the medium was changed to N2-supplemented MEM, which had been incubated for 3 days on a glial feeder layer before.

### Immunocytochemistry

Cells were seeded on poly-L-lysine-coated glass coverslips 24 h prior to treatment or transfection. Cells were fixed with 4% paraformaldehyde (PFA) (Applichem) in PBS for 10 min. After washing, cells were incubated for 30 min in 5% (v/v) ChemiBLOCKER (Millipore) solution containing 0.5% Triton X-100 for permeabilization and blocking. All antibodies were diluted in 2.5% ChemiBLOCKER solution containing 0.25% Triton X-100. Cells were incubated with primary antibodies for 1 h. After washing, Alexa Fluor 488- or 568-conjugated secondary antibodies (1:500; Life Technologies) were added for 1 h. DAPI (1 μg/ml) was used to stain cellular nuclei. All incubations were performed at room temperature (RT). Coverslips were mounted on microscopic slides using ProLong Gold Antifade Reagent (Invitrogen).

The cultured neurons were fixed after 4 days *in vitro* (DIV) with in PBS with 4% PFA and 4% sucrose for 15 min, quenched in 50 mM NH_4_Cl for 10 min and extracted with 0.1% Triton X-100 for 3 min. After washing, neurons were blocked for 1 h at RT with 2% fetal bovine serum (Gibco), 2% bovine serum albumin and 0.2% fish gelatin in PBS. Subsequently, cells were incubated with primary and secondary antibodies in 10% blocking solution for 1 h each at RT.

### Oligo(dT)-fluorescence *in situ* hybridization

HeLa cells were exposed to arsenite stress, washed once with PBS and fixed with 4% PFA for 10 min. Cultured neurons were fixed PBS with 4% PFA and 4% sucrose for 15 min. Cells were washed three times with PBS and then permeabilized with 0.2% Triton X-100 for 10 min, washed twice in PBS, followed by a 5 min wash with 2× saline-sodium citrate (SSC). Cells were prehybridized in prehybridization solution (Sigma-Aldrich) for 30 min. This was followed by hybridization in 4× SSC containing 50% formamide, 10% dextran sulphate, 1% BSA, 0.5 mM EDTA and 100 nM Cy3-oligo(dT)^30^ probe, overnight at 37°C in a dark humidified chamber. The following washing steps were applied: twice for 5 min in 2× SSC at RT, twice for 15 min in 2× SSC at 37°C, 10 min in 2× SSC at RT, 10 min in 0.2× SSC at RT. This was followed by immunocytochemistry starting with the blocking step.

### Confocal microscopy

Microscopy was performed using Zeiss LSM 780 laser scanning confocal microscope with ZEN 2010 software (Zeiss, black edition), equipped with lasers at 405, 488, 561 and 633 nm. Images were captured using 40× and 63× oil objectives. Images of all coverslips from one experiment were acquired using identical settings. Maximum intensity projections from *z*-stacks were generated in Fiji software (Schindelin et al., 2012). Fiji was used to adjust the brightness, contrast crop images and create scale bars.

### Quantification of stress granules and neuronal granules

Average number and mean area of cytoplasmic stress granules in HeLa cells were quantified using a customized pipeline in CellProfiler 3.0.0 (Carpenter et al., 2006). For all SG quantification experiments, between 200 and 365 cells per condition were counted from at least 25 random fields of view at 63× magnification. For rescue experiments, between 55 and 100 transfected cells were counted from at least 20 random fields of view.

In brief, nuclei were segmented using the DAPI signal. Whole cells were segmented as secondary objects, by extrapolating a specified distance from the edges of the nuclei based on the Alexa Fluor 488-TIAR signal. The punctate structures in the Alexa Fluor 488 channel were enhanced and detected as ‘Speckles’ with a feature size of 30 pixels in diameter. These enhanced speckles were annotated as stress granules. Only the granules in the cytoplasm region were quantified. Finally, feature count and mean feature area for stress granules were calculated and were exported for statistical analysis with R and Microsoft Excel. We analyzed the data from three repeat experiments using the Generalized Linear Model (GLM) and accounted for a confounding effect of the experimental data. For the average SG count per cell, the quasi-Poissonian regression model, and for the mean SG area per cell, the Gamma regression model were used. Graphs were plotted with GraphPad Prism 5.0.

For determining FMRP and OTUD4 colocalization, maximum intensity projections were used. Images were smoothened by applying a Gaussian filter (Fiji). Granules were separated from the background by adjusting the threshold of the smoothened image, followed by detection of local maxima. Clumped particles were separated by applying the watershed algorithm. By using the ‘Analyze particle’ plugin, OTUD4-positive granules were detected. The average fluorescence intensity of FMRP was measured in each OTUD4 granule with the ‘measure’ plugin in the region of interest (ROI) manager. A stringent cut-off value for FMRP fluorescence intensity was set to filter out the noise.

### Live-cell imaging

Live-cell imaging was performed with a GE DeltaVision Elite microscope (GE Healthcare, Life Science) running softWoRx 7.0 software. Images were captured using a 60× oil objective with 1.42 NA, and excitation bandpass at 542/27 nm for mNeonGreen–SMN1 and 597/45 nm for mOrange2–OTUD4. Time course images were captured at 500 ms intervals up to 4 min. Cells were analyzed at DIV4. While imaging, cells were maintained in a humidified 5% CO_2_ incubator at 37°C. All images were deconvolved using the standard softWoRx deconvolution algorithm. Fiji software was used for the following image analyses: the trajectories of moving granules were generated with MTrackJ plugin. The kymograph from the region of interest was generated using KymoResliceWide plugin. The ‘Straighten’ tool was used to straighten the neurites. Granule mobility over a period of 4 min was analyzed from kymographs using the ‘Velocity Measurement Tool’ plugin. Granules with maximum displacement (dx) >4 µm were considered as moving, with a dx <1 µm as stationary. Granules with dx values between 1 and 4 µm were scored as oscillating.

### Preparation of cell lysates and immunoprecipitation

Whole-cell lysates were prepared in lysis buffer (125 mM Tris-HCl pH 7.5, 150 mM NaCl, 1% Triton X-100, 10% glycerol, 1 mM EDTA, Complete protease inhibitor cocktail and 1 mM DTT). The protein concentration of lysates was determined by Bradford Assay (Bio-Rad).

For co-IPs, 10-cm dishes of HEK293T cells were transfected as indicated. After 48 h, cells were lysed with lysis buffer (see above). For RNase A treatment, cleared lysates were incubated with or without 50 μg/ml RNase A (Thermo Fisher Scientific) at 37°C for 15 min. IPs were carried out with anti-HA-coupled agarose beads (Sigma), anti-FLAG M2 affinity beads (Sigma) or GFP-Trap beads (Chromotek) as indicated. The beads were washed four times with 10 mM Tris-HCl pH 7.5, 150 mM NaCl, 0.1% Triton X-100 and 5% glycerol, and bound proteins were eluted with 0.1 M glycine, pH 2.5. Endogenous co-IPs were performed from three 10-cm dishes per condition, using protein A/G agarose beads (Santa Cruz Biotechnology) and custom-made anti-OTUD4 antibody or rabbit anti-HA antibody (Santa Cruz Biotechnology) as negative control.

### Western blot analysis

Immunoblot analysis was performed using standard protocols using horseradish peroxidase (HRP)-conjugated secondary antibodies (Biozol). Proteins were detected using WesternBright chemiluminescent solution (Advansta) or SuperSignal West Femto reagent (Thermo Fisher Scientific). Images were acquired on a ChemiDoc MP Imaging System (Bio-Rad). Band intensities were quantified with Fiji software.

### SUnSET assay

To monitor nascent translated proteins, the assay was performed as previously described (Schmidt et al., 2009). In brief, cells were treated with fresh medium containing puromycin (10 μg/ml) for 10 or 15 min 48 h after siRNA transfection. Caspase inhibitor z-VAD-fmk (20 µM) was added for 24 h prior to puromycylation. Cells were collected for western blot analysis.

### Preparation of mouse brain lysate

Mouse brain lysate from cortex or cerebellum of 6–8-week-old C57BL/6 mice was prepared as described in Schwintzer et al. (2019). All animal experiments were performed according to approved guidelines.

### HA affinity purification

HEK293T cells were transfected with pN3HA-OTUD4 (18 10-cm dishes) or control vector (15 10-cm dishes). At 48 h post transfection, cells were lysed for 1 h in ice-cold lysis buffer (20 mM Tris-HCl pH 8, 150 mM NaCl, 1% Triton X-100, Complete Protease Inhibitor Cocktail, 1 mM DTT and PhosSTOP phosphatase inhibitor). Lysates were incubated with HA-magnetic beads (Pierce) overnight at 4°C. Afterwards, beads were washed twice with low-salt buffer (20 mM Tris-HCl pH 8, 100 mM NaCl and 0.1% Triton X-100), twice with high-salt buffer (20 mM Tris-HCl pH 8, 150 mM NaCl and 0.1% Triton X-100), and again twice with low salt buffer. HA–OTUD4 immobilized to beads was then incubated with ∼5 mg of protein from mouse cortex or cerebellum lysate at 4°C for 5 h. Beads were washed four times in PBS with 0.5% Triton X-100 and subsequently incubated in Laemmli sample buffer for 5 min at 95°C. The HA-IP eluates were separated on 4–12% Bis-Tris gradient gels (NuPAGE, Life Technologies) and stained with SimplyBlue Coomassie G-250 SafeStain (Invitrogen). Gel slices were excised for analysis by mass spectrometry.

### Mass spectrometric analysis

Mass spectrometry and data analysis was performed as in Schwintzer et al. (2019). Proteins that were identified in at least two out of three experiments per tissue and were enriched ≥30 fold over control samples (HA-affinity purification from HA-vector-transfected cells; pulldown from mouse brain lysate) (based on peak area) were considered putative interactors.

### Oligo(dT) pulldown assay

mRNA-isolation was carried out using Dynabeads oligo(dT)25 according to the manufacturer's instructions (Invitrogen). HEK293T lysate was divided equally in three tubes for mRNA isolation. RNaseOUT ribonuclease inhibitor was added to lysis buffer (100 units/ml) and washing buffers (50 units/ml; not used in RNase A-treated samples). mRNA immobilized to beads was either washed with wash buffers A and B [10 mM Tris-HCl pH 7.5, 0.15 M LiCl, 1 mM EDTA, 0.1% LiDS (LiDS is not present in buffer B)] or was washed with wash buffer A and then treated with or without RNase A (100 µg/ml, Thermo Fisher Scientific) in wash buffer B for 15 min at 37°C. Dynabeads treated at 37°C were resuspended once more in wash buffer B. Proteins bound to the immobilized mRNA were eluted by incubating the beads in Laemmli sample buffer for 5 min at 95°C.

### RNA-IP and RT-PCR

HEK293T cells were transfected with pCMV-3Tag-6-OTUD4. Cells were UV-crosslinked (200 mJ/cm^2^, CL-1000 Ultraviolet Crosslinker, UVP) 48 h after transfection and resuspended in lysis buffer (20 mM Tris-HCl pH 7.9, 20% glycerol, 0.1 M KCl, 0.2 mM EDTA, 2.5 µl/ml RNaseOUT, 0.5 mM DTT and Complete protease inhibitor cocktail). To improve lysis, the cell suspension was snap frozen in liquid nitrogen and thawed at room temperature. Cleared supernatant was incubated overnight at 4°C with anti-FLAG M2 affinity gel or mouse IgG-agarose (both Sigma), and blocked with 2 mg/ml BSA. The beads were washed four times with lysis buffer and resuspended in lysis buffer. Contaminating DNA was digested with RNase-free DNase I (100 units/ml, OMEGA bio-tek) for 30 min at 37°C. To elute the RNA bound to immobilized FLAG–OTUD4, beads were treated with Proteinase K (0.7 mg/ml, Life Technologies) for 30 min at 37°C. The RNA was then extracted with phenol–chloroform and precipitated with ethanol. The RNA concentration was measured by using a NanoDrop (Thermo Fisher Scientific). For experiments with subsequent reverse transcription, the following more stringent buffers were used: lysis, 150 mM NaCl, 50 mM Tris-HCl pH 7.5, 0.5% NP-40, 2 mM EDTA, 2.5 µl/ml RNaseOUT, 0.5 mM DTT, Complete Protease Inhibitor; washing, 2× in lysis buffer and 2× in 500 mM NaCl, 50 mM Tris-HCl pH 7.5, 2 mM EDTA, 0.5% NP-40, 0.5 mM DTT and 1.25 µl/ml RNaseOUT.

Purified RNA was used as template for reverse transcription and amplification, carried out with the OneStep RT-PCR Kit (QIAGEN). Primer sequences were OTUD4 fwd, 5′-GGGATTTTGCCTTATGCTGA-3′ and OTUD4 rev, 5′-ACATGGGGCAAGAGTTGAAG-3′.

### *In vitro* transcription and biotinylation of RNA

RNA was *in vitro*-transcribed from a pCDNA3-3xHA-GFP expression construct downstream of a T7 promotor using the MegaScript™ T7 Transcription kit (Invitrogen) according to manufacturer's instructions. RNA was biotinylated during transcription using Biotin-11-UTP (Jena Bioscience) and UTP in a ratio of 1:5.

### *In vitro* RNA-binding assay

Expression of GST-tagged OTUD4 was induced in *E. coli* BL21 overnight at 18°C by 0.1 mM isopropyl β-d-1-thiogalactopyranoside in LB medium. Bacteria were lysed by sonication in lysis buffer containing 50 mM Tris-HCl pH 8, 100 mM NaCl, 1 mM EDTA, 1.4% N-lauroylsarcosine sodium salt, 1 mM DTT and Complete protease inhibitor cocktail. After the addition of one volume of 10 mM Tris-HCl pH 8, 150 mM NaCl, 1 mM EDTA and 2% Triton X-100, cell debris were spun down. The lysate was incubated with glutathione–Sepharose 4B (GE Healthcare) for 2 h. GST–OTUD4 immobilized to glutathione–Sepharose was washed three times with wash buffer (20 mM Tris-HCl pH 8, 100 mM NaCl, 1 mM EDTA and 0.1% Triton X-100) and once with assay buffer (10 mM Tris-HCl pH 7.5, 150 mM NaCl, 0.1% Triton X-100, and 5% glycerol). Recombinant GST was incubated with glutathione–Sepharose in lysis buffer for 1 h and was washed once with wash buffer and twice with assay buffer. Immobilized GST, GST–OTUD4, or glutathione–Sepharose were incubated with or without biotinylated RNA for 1 h at 4°C in assay buffer with 1 mM DTT (100 units/ml RNaseOUT ribonuclease inhibitor in samples with RNA, 50 units/ml in washing steps). Samples were washed three times with assay buffer and incubated with or without 2 µg/ml streptavidin-Alexa Fluor 430 (Thermo Fisher Scientific) for 30 min at 4°C. Samples were washed three times with assay buffer and transferred into a 96-well plate. Fluorescence intensity was measured using a Tecan Infinite^®^ F200 PRO plate reader. The background fluorescence of glutathione–Sepharose was deducted.

### Poly(A)^+^-RNA preparation

Poly(A)^+^-RNA was purified from equal numbers of siRNA control or siRNA OTUD4-transfected HeLa cells 48 h after transfection, using Dynabeads Oligo (dT)25 (Thermo Fisher Scientific) according to manufacturer's instruction.

### Polysome fractionation

Non-confluent HeLa cells were either treated with 0.5 mM sodium arsenite for 30 min or with 100 µg/ml cycloheximide for 15 min. Cells were lysed in a buffer of 10 mM Tris-HCl pH 7.4, 100 mM NaCl, 10 mM MgCl_2_, 1% Triton X-100, 1 mM DTT, Complete protease inhibitor cocktail and RNaseOUT ribonuclease inhibitor. After the addition of 0.3 µg/µl heparin, lysates were centrifuged through 15–50% (w/v) sucrose gradients for 110 min at 37,000 rpm in a SW 41Ti rotor (Beckmann Coulter). Each gradient was collected in nine fractions and each fraction was divided into two samples for RNA extraction and protein precipitation, respectively. RNA was extracted using phenol–chloroform, precipitated with ethanol and separated by agarose gel electrophoresis. Proteins were precipitated with ethanol, resuspended in Laemmli sample buffer, and analyzed by immunoblotting.

## Supplementary Material

Supplementary information
