# Supplementary material for: New roles for the de-ubiquitylating enzyme OTUD4 in an RNA–protein network and RNA granules
Source: J Cell Sci. 2019 Jun 17;132(12):jcs229252. doi: 10.1242/jcs.229252 (PMC6602300; doi:10.1242/jcs.229252)
Supplement: Supplementary information [file joces-132-229252-s1.pdf]

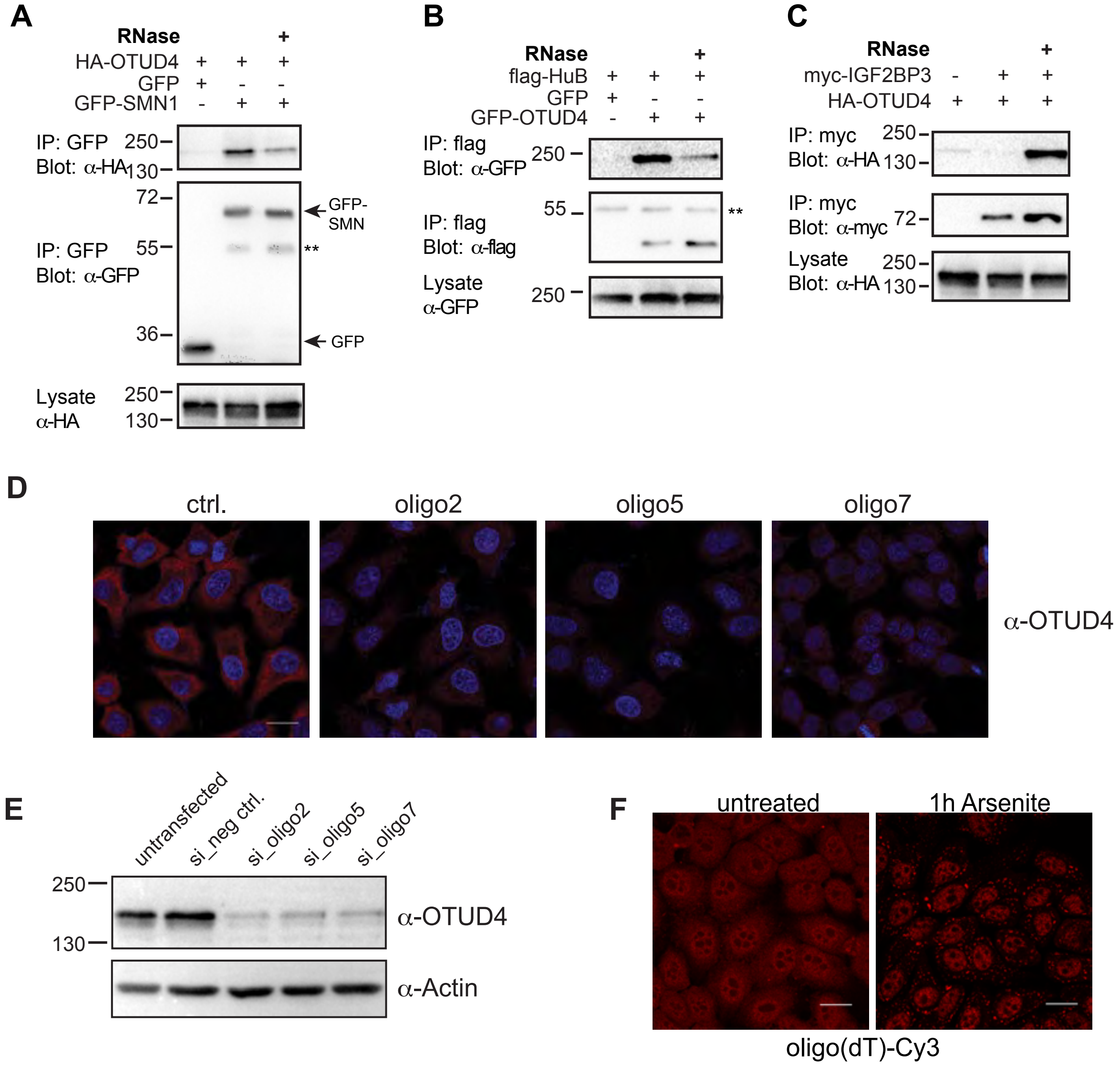

Journal of Cell Science • Supplementary information

Figure S1

A-C) Reversed co-IPs, related to Figure 1C-E. HEK 293T cells were transfected as indicated. Lysates were incubated at 37°C for 15 min in the absence or presence of 50 µg/ml RNase A. GFP-SMN1 (A), flag-HuB (B) or myc-IGF2BP3 (C) were precipitated and co-purification of HA- or GFP-tagged OTUD4 was detected by western blot. Treatment with RNase A (third lane of each panel) reduced or abolished the interaction of OTUD4 with SMN1 and HuB. In contrast, myc-IGF2BP3 was precipitated more efficiently following RNase treatment and OTUD4 co-precipitation could only be detected under RNase conditions. ** denotes antibody cross reaction with IgG heavy chain. A representative of at least 3 independent experiments is shown for each IP.

D) Hela cells were transfected with siRNAs against endogenous OTUD4 as indicated for 48 h and immunostained with antibody for OTUD4. Confocal images demonstrate clear knockdown of OTUD4 and specificity of the anti-OTUD4 antibody.

E) Western blot analysis of lysates from Hela cells treated with siRNAs against OTUD4 as in D).

F) Hela cells were arsenite-treated (0.5 mM) for 1 h or left untreated. FISH was carried out with Cy3-labelled oligo(dT) (red). Scale bar=20 µm.

Journal of Cell Science • Supplementary information

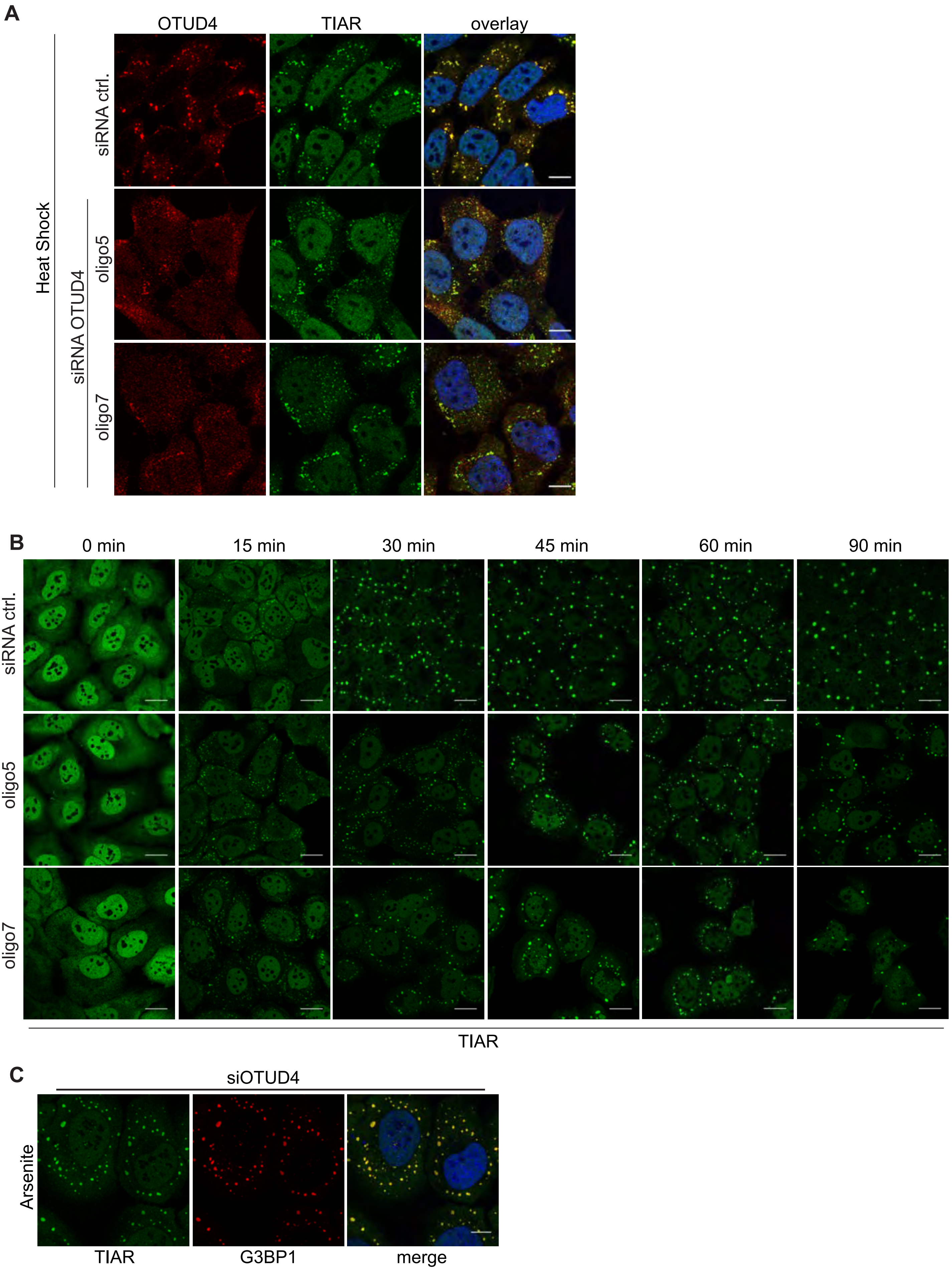

Journal of Cell Science • Supplementary information

Figure S2

A) (Related to Figure 5A). Knockdown of OTUD4 decreases size of heat shock-induced SGs and increases SG number. OTUD4 was depleted with siRNA in Hela cells. After 48 h, cells were heat shocked (46°C, 1 h) and subsequently stained for OTUD4 (red) and TIAR (green), scale bar 10 μm. The experiment was performed 2 times.

B) (Related to Figure 5A): Time course analysis of SG formation in control cells and OTUD4-knockdown cells. Cells were treated with 0.5 mM arsenite for the indicated times and stained for TIAR. The experiment was performed 3 times.

All scale bars=10 μm.

C) (Related to Figure 5A) OTUD4 knockdown affects TIAR- and G3BP1-positive stress granules upon arsenite treatment (0.5 mM, 35 min). OTUD4 was depleted by siRNA in Hela cells and stress granules were induced by arsenite treatment. Cells were co-stained for TIAR (green) and G3BP1 (red). The knockdown efficiency was confirmed by OTUD4 and TIAR co-staining in a parallel experiment (not shown).

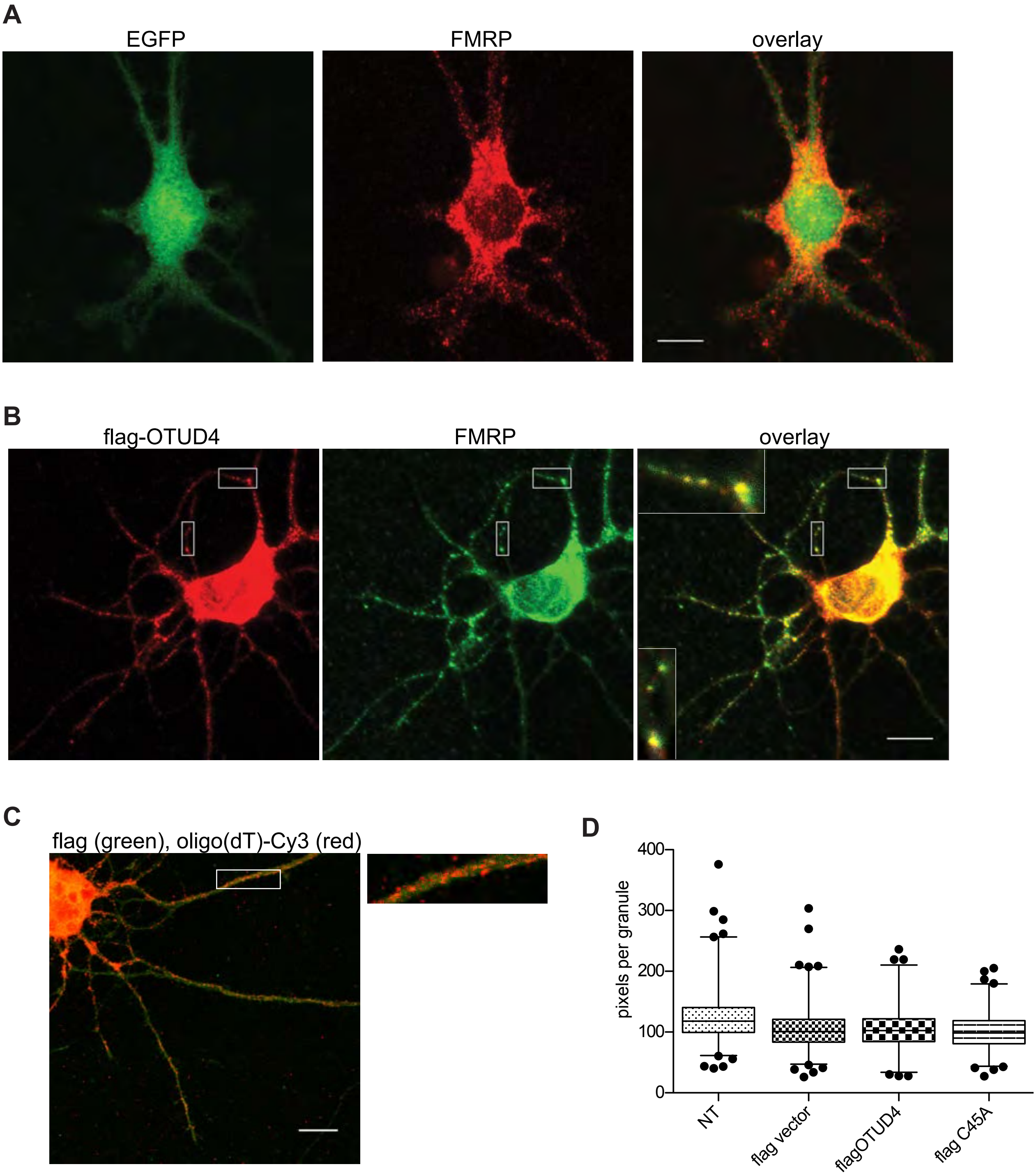

Journal of Cell Science • Supplementary information

Figure S3

A) Primary rat hippocampal neurons were transfected with EGFP and stained with anti-FMRP antibody (red) at DIV4 (scale bar=10 µm). This EGFP control does not show neuronal granules.

B) Rat hippocampal neuron, transfected with flag-OTUD4, stained with anti-flag antibody and anti-FMRP antibody. Flag-OTUD4 resides in neuronal granules and partially co-localizes with FMRP. Scale bar=10 µm.

C) (Related to Figure 4B) Rat hippocampal neuron, transfected with flag control plasmid (empty vector). *In situ*-hybridization with Cy3-oligo(dT) probe visualizes mRNA (red), co-stained with anti-flag antibody (green, weak diffuse staining). A magnification of the boxed region is shown. The oligo(dT) signal is comparable between control (this figure) and flag-OTUD4-transfected cells (Figure 4B), indicating that OTUD4 overexpression does not induce aberrant RNA-granule formation. Scale bar=10 µm.

D) Influence of transfection and OTUD4 overexpression on mean stress granule area. Hela cells were transfected as indicated or left non-transfected (NT). Stress granule formation was induced by arsenite treatment (0.5 mM, 30 min) and the average size per granule was determined as in Figure 5C and 5F. The transfection procedure does mildly reduce the granule area but OTUD4 overexpression does not increase granule size compared to empty vector transfection, ruling out that rescue effects shown in 5F were caused by overexpression per se.

Journal of Cell Science • Supplementary information

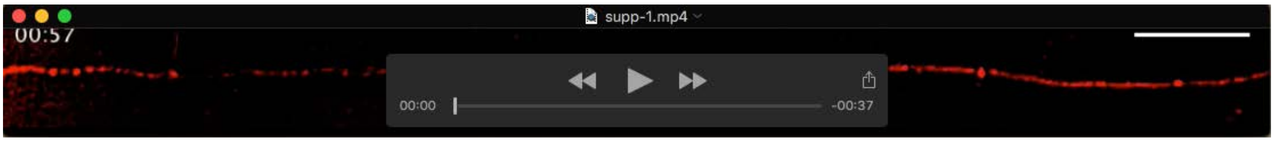

Movie 1

Video of mOrange2-OTUD4-granules in DIV4 rat hippocampal neurons.

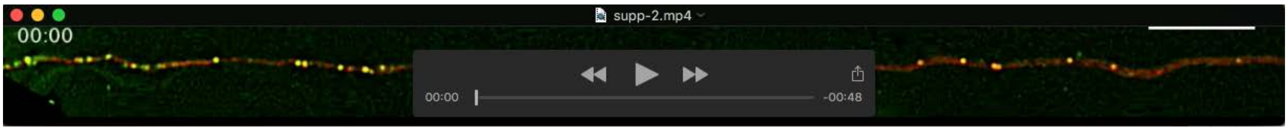

Movie2

Video of mOrange2-OTUD4 and neonGreen-SMN1 granules in DIV4 rat hippocampal neurons.

Table S1
List of OTUD4 interactors and legend for Table S2 and S3

Click here to Download Table S1

Table S2
Mass spectrometry data, Cerebellum data set

Click here to Download Table S2

Table S3
Mass spectrometry data, Cortex data set

Click here to Download Table S3

Journal of Cell Science • Supplementary information
